# Predictors and Barriers to Post Abortion Family Planning Uptake in Hai District, Northern Tanzania: A Mixed Methods Study

**DOI:** 10.24248/eahrj.v5i2.671

**Published:** 2021-11-15

**Authors:** Benjamin Asubiojo, Peter E. Ng’wamkai, Benjamin C. Shayo, Rose Mwangi, Michael J. Mahande, Sia E. Msuya, Eusebious Maro

**Affiliations:** a Department of Obstetrics and Gynecology, Kilimanjaro Christian Medical Center, Moshi Tanzania; b Kilimanjaro Christian Medical University College (KCMUCo), Moshi, Tanzania; c Department of Epidemiology & Biostatistics, Institute of Public Health, Kilimanjaro Christian Medical University College (KCMUCo), Moshi, Tanzania; d Department of Behavioural and Social Science, Institute of Public Health, Kilimanjaro Christian Medical University College (KCMUCo), Moshi, Tanzania

## Abstract

**Introduction:**

Post Abortion Care (PAC) encompassing family planning counselling and contraception provision is a key strategy to reducing maternal morbidity and mortality especially in countries with restrictive abortion laws. Various factors affect the uptake of PAC modern family Planning (FP) in different settings. This study aimed at determining the prevalence, assessment of factors and barriers to PAC modern FP uptake in Hai district, Northern Tanzania

**Methods:**

A mixed-methods study was conducted using an explanatory sequential design. Exit interviews using questionnaires was conducted among 189 women. In-depth interviews were conducted with 26 healthcare providers (HCPs) and 28 women who received PAC in Hai district hospital, Machame hospital and Moshi Specialists health centre in Hai district. Quantitative data was analysed using a Statistical Package for Social Science (IMB SPSS Statistics for Windows version 20.0 (SPSS Inc., Chicago, Ill., USA)). Bivariate and multivariable analyses were applied to estimate the predictors of uptake of PAC modern FP. Thematic content analysis was employed to explore barriers to uptake of post-abortion modern family planning.

**Results:**

The prevalence of uptake of modern family planning following PAC was 59/189(31.2%). 56% of the 189 women who received PAC did not receive counselling services on family planning. Marital status and partner's support were predictors of PAC modern family planning uptake (*p=.007* vs. *p= <.05,* respectively).

Misinformation and misconception about modern contraceptives, lack of knowledge and fear of side effects were reported to be the major barriers to uptake of post-abortion family planning. Most women reported to have not received comprehensive family planning information from the HCPs. On the other hand, HCPs perceived their poor counselling skills as the barrier to post-abortion family planning uptake. This study observed poor coordination of PAC services within each visited facility and this was linked to women leaving the facility without family planning counselling and/or contraceptives provision.

**Conclusion:**

Suboptimal modern family planning counselling during PAC contributes to the low uptake of contraceptives methods in this setting. Strategies are needed to improve PAC modern family planning services uptake. Strategies such as; provision of counselling skills to HCPs with comprehensive information targeting local contextual misconception and promoting PAC provision as a one-stop service.

## BACKGROUNDS

Globally, spontaneous and induced abortion remains a major public health concern. Approximately, 25 million unsafe abortions take place worldwide each year, with majority happening in developing countries with restrictive laws against abortion.^1^ The complications related to unsafe abortions can be fatal and contribute up to 13% of maternal deaths worldwide^2,3^, with Africa contributing up to 29% of these abortion-related deaths.^1^ In Tanzania, 15% of all pregnancies ended in abortion in 2013^4^ and unsafe abortion accounts for 25% of all maternal deaths.^5^ Post-Abortion Care (PAC) which encompasses emergency treatment of complications due to incomplete abortion or miscarriages, family planning counselling and services provision as well as linkage to other reproductive health services is a key strategy to reducing abortion-related morbidity and mortality, especially in countries with restrictive laws against abortion where the majority of unsafe abortion occurs. According to Tanzania family planning guidelines, family planning services should be provided at the same time and location where women receive PAC services.^6^

The post-abortion period constitutes a unique opportunity to introduce family planning counselling and optimise chances of family planning uptake and prevent unwanted pregnancy.^7,8^ Thus, family planniing counselling and provision of contraception is one of the elements of comprehensive post-abortion care.^9,10^ Studies conducted in Italy and Nigeria have shown that modern family planning uptake following PAC could be as high as 65% and 79% respectively, whereas, studies conducted in Tanzania and Brazil reported uptake to be 90% and 97% respectively when effective family planning counselling and contraception provision is offered immediately as part of PAC.^8,11–13^ However, even though emergency treatment may be delivered at satisfactory levels in some countries with restrictive abortion laws, the full package of family planning counselling, education and methods provision is often not provided before the women leave the health facility where they received PAC, missing the opportunity to prevent future unwanted pregnancies plus associated morbidities.^14^

Several factors influence the provision of family planning methods following PAC for both women and Health Care Providers (HCPs) perspectives. Studies conducted in Uganda, Nepal and Brazil have reported that women's contraceptive decision-making autonomy influences uptake of contraception after PAC.^15–17^ Age has also been associated with uptake of modern family planning following PAC, with inconsistent findings. A cohort of 18,688 PAC clients in Tanzania, reported older age (>35) to be less likely to take up contraception following PAC.^18^ However, a study was conducted in Ghana plus other studies conducted in 10 countries in Asia and Sub Saharan Africa reported young women of ages 10 to 19 years, to be less likely to accept modern contraceptives following PAC than others^19,20^ Health system factors also influence post-abortion uptake of contraceptive methods. These include: clinic logistics that impact the provision of standard family planning counselling and provision, limited contraceptive choices, few numbers of trained providers, multiple clinic visits and poor integration with other existing health services.^21–24^

Contraceptive uptake and use is said to be high when PAC family planning services are provided. However, there is limited data on the uptake of family planning following PAC from countries with restrictive laws towards induced abortion like Tanzania. In the USA, immediate PAC contraception provision, such as placement of an Intrauterine device (IUD) has shown a significant decrease in unintended pregnancy in the year following abortion as compared to delayed insertion.^2^ In low resource countries, the uptake of immediate PAC modern family planning methods such as IUD and implant might be hampered due to several healthcare system-related challenges such as lack of enough staff with skills to provide these methods. In order to overcome the barriers and improve the quality of PAC, there is need to understand the challenges faced by health care providers and women during the provision of PAC family planning services.

Although Tanzania's family planning agenda is geared to make family planning methods available at all levels of care including during PAC^24^, its modern contraceptive prevalence rate is still low at 32%^25^ compared to the national target of 45%.^26^ Therefore, provision of family planning counselling during PAC provides an opportunity to increase family planning uptake among women receiving PAC. Challenges faced by both women receiving PAC family planning services and healthcare providers need to be better understood so as to overcome the barriers and improve the quality of family planning services provision during PAC. There are few studies that have assessed the healthcare system factors that affect women's uptake of PAC family planning.^27,28^

This study aimed at determining prevalence of and predictors to uptake of modern family planning following PAC in Hai district. The study also explored barriers to uptake and provision of modern family planning methods in the rural area of Kilimanjaro, Hai district, Tanzania. The findings of this study will guide the healthcare system to strengthen PAC family planning service provision and design better strategies to overcome the existing challenges.

## METHODS

### Study Design and Setting

We deployed a mixed-methods study using an explanatory sequential design, whereby quantitative data was collected first; then qualitative data was gathered to interpret the quantitative findings. Health facility-based quantitative data was collected among women receiving PAC, to determine the prevalence of and predictors of uptake to PAC family planning. This was followed by qualitative interviews of Health Care Providers (HCPs) and women who did not take PAC modern family planning to determine barriers to uptake of PAC family planning. This study was conducted in 3 health facilities in Hai district, Northern Tanzania from August 2017 to May 2018.

Hai district is located in rural Kilimanjaro region in Northern Tanzania. According to Tanzania's National Census of 2012, the population of Hai district was estimated to be 210,533 people. The unmet need for family planning among married women in Kilimanjaro region is 17.7%, below the national average which is 22%. The unmet need for family planning among unmarried women in Kilimanjaro stands at 11.1% compared to the national average of 17.7% among young women aged 20 to 24 years of age.^29^

Tanzania health care system is organised into 4 levels; dispensary, health centre, district hospital and referral hospital in increasing orders of the population served.^30^ Hai district has a total of 62 health facilities. These include; 2 hospitals, 6 health centres, and 54 dispensaries. This study was conducted in 3 health facilities namely, Hai district Hospital, Machame Hospital and Moshi Specialist Health Centre. These facilities actively provide PAC services in Hai district. They also provide modern family planning counselling and services as part of PAC package. Modern family planning methods offered include; Intrauterine Contraception Device (IUCD), implant, injectable, oral contraceptive pills and condom.

### Study Population and Sample Size Estimation

All women residing in Hai district who were seeking PAC at study sites during the study period were eligible to participate in the study. The study population was of women who presented to the health facilities with symptoms and signs of abortion i.e. pregnant women at gestation age less than 28 weeks with vaginal bleeding, lower abdominal pain, conjunctival pallor, tender lower abdomen and were clinically diagnosed by a physician. Therefore, all women who attended the 3 health facilities for PAC during the study period were included. We excluded all women who were referred to higher health facility due to complications since they did not receive the complete PAC package at the study sites.

In addition, Health Care Providers (HCPs) providing PAC services and those working in family planning clinics in the selected health facilities were purposively selected. The selection of HCPs considered candidates who were present on-duty during the study period, considering the representation of all the carders i.e. doctors, nurses and midwives. The selection of HCPs also considered candidates with working experience of more than one year at their duty station. The HCPs were recruited from all service units where woman pass during PAC, including female wards, operating theatre, PAC room and family planning clinic. Health care providers who were absent during the study period were excluded.

The sample size for the quantitative interview was estimated based on the prevalence of PAC modern family planning uptake of 79.8% reported in 2016 by Onyegibule et al.^8^ and an acceptable marginal error of 5%. This gives a sample of 248 women. 10% of the sample size was added to take care for non-response rate. The final sample size became 273 women. However, the response rate was 89.5% (222/248).

Purposive sampling was used to select women who did not uptake modern contraceptives during PAC to participate in in-depth interviews. Age and parity guided the selection of these participants, based on the assumption that women aged 20 years and above, and/or women with parity more than two have more contact and exposure to healthcare system hence will have information regarding barriers to uptake of PAC services.

### Data Collection Methods and Tools

Women who presented to the 3 facilities with symptoms and signs of abortion were first examined by the local physician. The physician verbally consented to each woman who attended for PAC to allow an interview with the research assistants regarding PAC. On the daily basis, the research assistants were notified of any woman who has attended the health facility for PAC and had agreed to be interviewed. 3 research assistants, nurse and midwives were trained to assist with data collection. The research assistants evaluated each participant to see if they meet the set eligibility criteria. Participants who were eligible were invited into the study upon discharge(before leaving the health facility). Written informed consent was obtained from all participants. Data was collected through an Exit Interview (EI) in a private room face to face using an interviewer-administered questionnaire. The information collected from the participants included socio-demographic characteristics (age, address, religion, occupation, marital status and level of education), parity, gestation age at abortion estimated from the last normal menstrual period, fertility intentions, previous use of contraception, whether contraceptive counselling was offered and the contraceptive method taken.

Following EIs, women who did not uptake modern family planning methods were invited for an In-Depth Interview (IDI) to explore the barriers. Verbal consent was sought for and IDIs were conducted in a private room within the health facility. The IDIs among women explored their perception on the reason for non-use of PAC modern family planning, their knowledge and belief on modern family planning methods and their experience on the general PAC and family planning services offered at the health facility. These IDIs were conducted by a trained research assistant following a list of guided questions and the interview was audio-recorded. Similarly, HCPs providing PAC and those working in family planning clinics in the 3 facilities were also invited to participate in the study. Informed written consent was obtained before initiation of the IDIs. The IDIs were conducted by research assistants in a private room through a list of guided questions. The IDIs explored HCPs' views on barriers to PAC modern family planning use, HCPs skills, number of staff available, availability of medical supplies and health system-related challenges in the integration of PAC with other reproductive health services. All IDIs were conducted in Kiswahili language and each interview lasted approximately one hour. The sample size was attained after the saturation point was reached, at this point no new emerged themes were generated from the interviews.

### Data Analysis

Quantitative data was analysed using a Statistical Package For Social Science (IMB SPSS Statistics for Windows Version 20.0 (SPSS Inc., Chicago, Ill., USA)). For continuous variables, normality was checked using a histogram. Symmetrical variables were summarised using mean and Standard Deviation (SD), while asymmetrical variables were summarised using median and Interquartile Range (IR). Frequencies and proportions were used to summarise the categorical variables. Odds Ratios (OR) with 95% Confidence Interval (CI) for predictors of PAC modern family planning uptake were estimated using bivariate and multivariable logistic regression analysis. A p-value of less than 5% was considered significant.

Qualitative data was transcribed in Kiswahili language and then translated into English. An iterative process was used to analyse the data using thematic coding framework to assess all interviews' transcripts based on the IDI guides. To ensure inter-coder reliability, all the transcripts were coded by at least 2 of the authors and discrepancies were resolved through discussion. Since this part of the study was inductive in nature, quotations from the study participants were used to characterise issues and themes that emerged. The analysis also looked at patterns and associations of these themes. Themes that were illustrative were selected and summarised, focusing on the barriers to post-abortion modern family planning uptake and provision.

### Ethical Considerations

Ethical approval was obtained from Kilimanjaro Christian Medical College Research Ethics and Review Committee (CRERC), with clearance certificate number 2027. Permission to conduct the study was sought from the Kilimanjaro Regional Medical Officer, the District Medical Officer for Hai and heads of the facility in the 3 study sites. Before enrolment into the study, detailed information was provided and explained in Kiswahili language to participants. The right to withdraw or refuse participation in the study was made known to individual participants and only those who were willing and signed an informed consent form were included in the study. Anonymity of participants was maintained at all times by using identification numbers as opposed to using participants' names.

## RESULTS

### Quantitative Findings Uptake and Characteristics of the Study Participants

**FIGURE 1.**
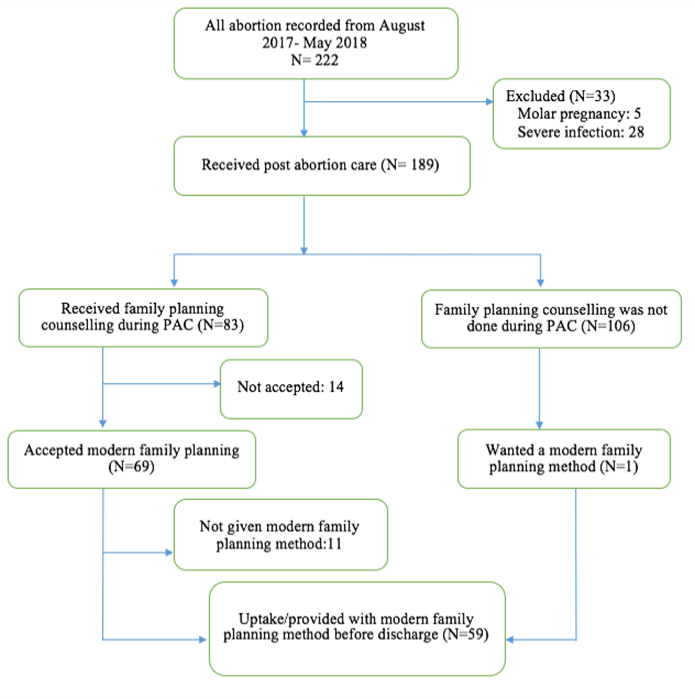
Flow Chart Depicting Counselling and Uptake of Modern Family Planning During PAC

A total of 222 women attended PAC in the 3 health facilities studied from August 2017 to May 2018 (Figure. 1). 33(14.8%) of these were referred to higher centres due to various conditions such as molar pregnancy (5) and severe infection (28) and these were excluded from the study as they did not receive the complete PAC package at the study sites. The remaining 189 women were eligible and agreed to participate in the study. During exit interviews of the 189 women, 83 (43.9%) reported having been counselled on modern family planning methods during PAC while, overall, 70(37%) of the participants demonstrated their intention for modern family planning use. Only 59 women were provided with their preferred modern family planning method before leaving the health facility on discharge, making the proportion of modern family planning method uptake during PAC to be 59(31.2%). Moreover, of the 70 women who agreed to PAC modern family planning following PAC, only 59(84%) were provided with their chosen method before leaving the health facility.

Of these 59 women who selected a method, implant was the most common method of choice 20(34%), followed by IUCD 17(29%) and Oral contraceptive pills 13(22%). Depo-provera injections and condoms were selected by 7(12%) and 2(3%) of the women respectively.

The characteristics of study participants and health facilities are shown in Table 1. The mean (Standard Deviation) age of 189 women was 29.0 (SD 6.5) years. Their age distribution ranged from 16 to 43 years. Majority 148(78.3%) were married/cohabiting, self-employed 144(76.2%) or had ever used family planning methods before the current pregnancy 124(66%). More than half 103(55%) of the participants were enrolled from Hai district hospital. Majority 166(88%) of the 189 women who received PAC reported that it was their first abortion. Majority 150(79.4%) were of second trimester abortions which the median gestation age (calculated from the last normal menstrual period) of the index pregnancy at which abortion occurred was 16 weeks, with the range of 7 to 26 weeks. Half 96(50.8%) of women had 2 or more living children, whereby the median number of living children was 2 (range: 0-8).

**TABLE 1:** Characteristics of the Study Participants (N=189)

Variables	n	%
**Age, years**		
<20	15	7.9
20–24	38	20.1
25–34	94	49.7
>35	42	22.2
**Marital status**		
Single/separated/divorced	41	21.7
Married/cohabit	148	78.3
**Level of education**		
None	7	3.7
Primary	113	59.8
Secondary	62	32.8
Higher education	7	3.7
**Occupation**		
House wife	17	9.0
Formal employed	25	13.2
Self-employed	144	76.2
Student	3	1.6
**Used FP before**		
Yes	124	65.6
No	65	34.4
**Length of hospital stay**		
1 day	90	47.6
2 or more	99	52.4
**Facility enrolled**		
Hai Hospital	103	54.5
Machame Hospital	60	31.7
Moshi Specialist	26	13.8
**GA at abortion**		
<12 weeks	39	20.6
12–28 weeks	150	79.4
**Number of children**		
None	32	16.9
One	61	32.3
≥2	96	50.8

### Socio-Demographic, Reproductive and Obstetrics Characteristics Associated With Uptake of Modern Family Planning During PAC

Findings from the univariate and multivariable logistic regression models are summarised in Table 2 and Table 3 respectively. In both univariate and multivariable analyses, women who were single or separated were significantly associated with higher odds of utilising post abortion care compared with married/cohabiting counterparts (OR: 2.33; 95% CI 1.14-1.76), (aOR: 2.80; 95%CI 1.32-5.91). It was also revealed that women who reported that their partners would support their decision to use contraception, were significantly associated with uptake of modern family planning during PAC compared to their counterparts in both univariate and multivariable analyses (OR: 1.87; 95%CI 1.00-3.49), (aOR: 2.22; 95%CI 1.15-4.28). Other socio-demographic characteristics were not significantly associated with uptake of PAC family planning.

**TABLE 2:** Socio-Demographic, Reproductive and Obstetric Characteristics Associated with uptake of Modern Family Planning during PAC (N=189)

Variable	N	Modern Family Planning Uptake (n=59)	Unadjusted OR (95%CI)
**Age(years)**			
≤24	53	21(39.6)	1.00
25+	136	38(27.9)	0.59(0.30-1.15)
**Marital status**			
Married/cohabit	148	40(27.0)	1.00
Single/divorce/separated	41	19(46.3)	2.33(1.14-1.76)
**Level of education**			
None/primary	120	38(31.7)	1.00
Secondary and above	69	21(30.4)	0.94(0.50-1.79)
**Occupation**			
Formal employed	25	5(20.0)	1.00
Other	164	54(32.9)	1.96(0.70-5.52)
**Used FP before**			
Yes	124	43(34.7)	1.63(0.83-3.19)
No	65	16(24.6)	1.00
**Regular partner**			
No	33	49(31.4)	1.05(0.466-2.381)
Yes	156	10(30.3)	1.00
**Partner support**			
No	97	24(24.7)	1.00
Yes	92	35(38.0)	1.87(1.00-3.49)
**Number of Abortion**			
1	166	54(32.5)	1.00
2+	23	5(21.7)	0.58(0.20-1.63)
**Number of children**			
None	32	13(40.6)	1.00
One	61	17(27.9)	0.56(0.23-1.39)
≥2	96	29(30.2)	0.63(0.28-1.45)
**GA at abortion**			
<12 weeks	39	8(20.5)	1.00
12-28 weeks	150	51(34.0)	2.00(0.86-4.66)
**Length of hospital stay**			
1 day	90	32(35.6)	1.00
2 or more	99	27(27.3)	0.68(0.37-1.26)
**Site of enrollment**			
Hai DH	106	37(35.9)	1.93(0.66-5.62)
Machame hospital	60	17(28.3)	1.42(0.46-4.45)
Moshi Specialist	23	5(19.2)	1.00

cOR: Crude Odds Ratio

**TABLE 3:** Final Model on Factors Associated with Uptake of Modern Family Planning during PAC (N=189)

Variable	aOR	(95% Cl)	*P-value*
**Marital status**			
Married/cohabit	1.00		
Single/separated/divorced	2.80	(1.32-5.91)	.007
**Partner support**			
No	1.00		
Yes	2.22	(1.15-4.28)	.017

aOR: adjusted odds ratio

Adjusted for age, marital status, partner support, and previous use of family planning

### Qualitative Findings

A total of 130 (68.8%) women did not uptake any modern method of family planning and were eligible to participate in In-Depth Interviews (IDIs) to explore more reasons and barriers to the use of PAC contraceptives. The saturation point was reached after 28(24 %) interviews. Of the 28, 18 women were enrolled from the 2 hospitals and 10 from the health centre. The age of 28 women involved in the IDIs ranged from 20 to 43 years and they were either married, cohabiting or single. All participants reported having heard of different types of contraceptives from Ante-Natal Clinic (ANC), co-workers, peers and family members.

A total of 26 HCPs of different cadres were enrolled for IDI; these included Medical Officers, Assistant medical officers, clinical officers, nurses and midwives providing PAC services at various points such as theatre, female surgical wards, maternity wards and those working at family planning clinics. HCPs were interviewed to explore barriers in provision and uptake of PAC modern family planning based on their individual perception and experience in the context of healthcare system-related challenges. HCPs interviewed included males and females with age range between 26 to 58 years. Out of the 26 HCPs; 6 were from the health centre and the rest were from the 2 hospitals.

### Women Perception on Barriers to Uptake of Post-Abortion Modern Family Planning

After engaging individual women after PAC, from the IDIs, a number of themes emerged. These included; Fear of side effects, misconception, myths and misinformation, gender power differences and partner support, and experience with health care providers in seeking family planning.

***Fear of Side Effects:*** Majority of the women participating in the IDIs expressed their fear of use of modern family planning methods. Most of them mentioned a wide range of side effects including; nausea, losing or gaining weight, prolonged menses, swelling of feet and infertility as indicated by these 2 participants:

*‘I once used pills and after a while, I was bleeding a lot then I stopped and used injections, and the situation got even worse- I was bleeding from day 1 to day 15, I was getting palpitations, I stopped and used implants for 3 years; it also caused palpitations and I stayed with it for 2 years and a half then I decided to go to the doctor to remove it, and since then I haven't used any method’* (43 years old, 6 living children).

*‘I carried pregnancy without knowing…. but I was using contraceptives before…. I saw bad side effects like swollen lower limbs (…) I decided to stop using them’* (40 years old, 3 living children).

***Misconception, Myths and Misinformation:*** The use of pills as a modern contraceptive was attributed to a range of complications that are not medically known to be side effects or to have an association with abortion. Although some women claimed to be on the pill, they had stopped due to potentially perceived complications. For example, one of the participants associated her nausea and abortion with the use of family planning pills.

*‘I do not know because I was using pills…. I didn't know if I use pills they will cause problems (abortion). When I use pills I get nausea, I feel like vomiting……I think those pills caused this problem (abortion)’* (29 years old, 1 living child).

Another participant had similar sentiments:

‘*I did not know I was pregnant…. am not even expecting to conceive…. I will continue to use the calendar; I do not want any contraceptives…. I think they (modern contraceptives) contributed or they were the cause of this abortion…… I think these contraceptives have some kind of poison’*

Participants cited lay sources of family planning information such as fellow women in the village.

*‘I got this information in the village from other women… Some say they used implants and wasn't good for them, some say injections are bad’* (30 years old with 2 living children)

***Gender Power Differences/Partner's Support***: Some participants showed willingness to space out children before the next pregnancy. However, factors such as the desire for children by the spouse/partner and lack of partner support, made it difficult for such participants to uptake any birth control method. The desire to space children despite lack of partner's support made some participants decide to use modern family planning methods secretly;‘…*He had asked me why am I not getting pregnant. But I couldn't tell him anything, if I tell him I was using contraceptives he would be so angry at me… he doesn't even understand what contraceptives mean’* (29 years, 1 living child).

### Healthcare Providers' Perception on Barriers to Uptake of Post-Abortion Modern Family Planning

Many factors were identified as barriers to uptake of PAC modern family planning, including lack of spousal support, misleading beliefs on contraceptives and the desire to get another pregnancy as soon as possible, especially for participants who are married or cohabiting with their partners without any child. In many cultures in the African settings, this helps the woman to establish herself in that relationship/marriage. Apparently, the reported barriers by HCPs matched those reported by women who participated in this study.

*‘Some of them really need a child so if you don't explain to her, she won't understand why she has to wait for some months. It is not a simple thing to tell a woman not to conceive for a couple of months, especially those who are married and they don't have children*’ (Nurse, 2 years' experience, family planning clinic).

Seeking HCPs' opinions on what could be done to give better care. All individual providers interviewed agreed to the need for capacity building and provision of regular training opportunities aimed at improving their skills. *‘…. more HCPs should be employed*…… *it is good to have regular update trainings'* (Assistant Medical Officer, 10 years' experience, female ward and theatre).

*‘I can try to talk to her [patient] on family planning……but I wish to get proper family planning training to improve myself’* (Nurse, 3 years' experience, female ward).

### Women's Perception on the Barriers to Provision of Post-Abortion Modern Family Planning Methods

#### Experience with Health Care Providers

There were diverging views on individual women experiences with the HCPs. Majority of the participants reported that the HCPs never discussed or mentioned post-abortion contraception to them during their stay at the facility for PAC. On the other hand, the other group of women described their experience as a piece of advice in favour or against a specific method. In some cases, it was clear that whenever contraceptives were provided, participants were given little information and choices to decide on the methods;

‘*I have used them [contraceptives] to prevent pregnancy… Implants, IUCD and pills…… so I decided to stop using any of them. Yes, now I do not know what to do… but I wish before a mother is given any contraceptive method, she should be counselled on available methods and be told if the method chosen is suitable for her or not’* (28 years old, one living child).

A newly married woman who had never used modern family planning method but desired to give space of 4 to 6 months to the next pregnancy shared her experience. She had never heard about modern contraceptives and as well did not receive counselling or given information about family planning methods during this time of receiving PAC;

*‘I have never used any family planning method…. I am going to rest for 4 to 6 months*…… *You [interviewer] are the first one to tell me about it [family planning] ……’* (23 years old, no child).

Another participant, married with 3 living children and not willing to ever become pregnant again wanted a permanent family planning method, however, HCP did not discuss of any family planning method with her and during her visit for PAC

*‘I do not want [pregnancy] at all, I am not ready for that may God help me…my plan now is to go home and rest for a while… I will come for tubal ligation’* (40 years old, 3 living children).

In-depth interviews clearly indicated that there was inadequate information given to patients about modern family planning methods. One of the participant's response demonstrates that uptake of family planning methods by women can greatly improve if HCPs provides adequate birth control information to patients.

‘*She [Nurse] mentioned IUCD ……and she advised me that it doesn't have problems and could be there for 12 years. I asked if there are times am supposed to come and see if the position of the IUCD is in the same place…… the nurse said there is no need, it doesn't have problem’* (43 years old, 6 living children).

### Healthcare Providers' Perception on Barriers to Provision of Post-Abortion Modern Contraceptives

Generally, HCPs reported that there were adequate family planning commodities; hence availability would not have been a barrier to uptake of modern contraception. However, during the in-depth interviews with the HCPs, 2 key themes emerged which posed barrier to uptake, namely; inadequate skills and lack of coordination and integration in the existing routine of family planning and those who had gone through post-abortion care.

When HCPs were asked whether counselling and provision of modern family planning was provided for post abortion mothers, in all the 3 facilities, there were varying responses to this question, i.e. while HCPs at the family planning clinic would say it's done in the ward, colleagues in the ward would say it is done at the family planning clinic. This showed disjointed and lack of coordination and integration in as far as providing comprehensive care is concerned. In all the 3 facilities, it was clear that the roles of HCPs in theatre, wards and family planning clinic towards PAC modern family planning counselling and methods provision is not well defined and hence there was deficient services and overlapping of duty as indicated by the HCPs response below; *‘Yes, [post abortion] patients are there, … we get one or two per month at Reproductive Health Clinic (RHC), but not many come here [RHC] because most of them get the service while still in the wards, and only a few come here. They get this information [about family planning] at the ward… That's why all the equipment is found at the ward*’. (Nurse, 8 years' experience, family planning clinic).

*‘After the procedure in theatre, the patient is transferred to the maternity ward for further care, after maternity, she goes to family planning clinics'* (Enrolled Nurse, 4 years' experience, theatre).

*‘No, we don't counsel them here [female ward] …. After she is discharged by the doctor from here [female ward], we direct them to RHC for family planning counselling and provision’* (Nurse, 10 years' experience, female ward).

This sequence of care makes service delivery very complex and confusing to the patients. Hence, despite high numbers of women that are treated for various abortion complications, family planning counselling and methods provision is given only to a few.

The study further explored the perceived knowledge and skills of HCPs to carry out their duties when it comes to caring for PAC patients. Almost all HCPs enrolled in the IDIs had no formal training in the provision of PAC.

Nearly, all the HCPs, college training where PAC was part of the topics taught was the only orientation HCPs considered as skills for PAC. Common sentiments could be summarised by these 2 HCPs;

‘*I had a short training on family planning at KCMC* for a week…. *Some of us do go for those trainings, but I have never attended for any workshop meant to upgrade my skills'* (Nurse midwife, 8 years' experience, family planning clinic).

*‘I have never had any training in post-abortion care except general knowledge during my training as a nurse so many years ago’* (Nurse, 5 years' experience, female surgical ward).

HCPs further reported that while a few staff members were privileged to attend available training dedicated to PAC once in a while, the difficulty in sharing information/knowledge acquired was quite obvious as expressed by a nurse who was stationed in the theatre;

*‘… if only a few staff members went for training, it would serve us if they came back and trained the other members about the updates so that we are all well informed’* (Nurse, 2 years' experience, theatre).

Some HCPs stated that, training was no longer being offered as regularly as in the past.

*‘We used to have them [training] in the past, but nowadays they are not there, so if we could have them that would be great’* (Nurse Midwife, 12 years' experience, family planning clinic).

Inadequate knowledge and skills on family planning counselling was also realised. HCPs reported recommending specific modern family planning methods for woman instead of providing proper counselling by provinf adequate information about all the available methods to give opportunity to the woman to choose what suits her best.

*‘Yes you select for her and you must explain to her why you chose that method for her*’

(Nurse, over 20 years' experience, family planning clinic).

#### Availability of Modern Family Planning Commodities

When asked whether they ran out of stock for modern family planning commodities, HCP participants from all - the 3 facilities acknowledged that the units were well supplied with required commodies. However, they reported reduced number of supplies for certain family planning methods. This is what a nurse at reproductive health clinic stated;

*‘Yes, family planning commodities are available…. we are not completely out of stock before they finish, we order for new ones…. therefore, it has never happened that we are without contraceptive methods'* (Nurse, 8 years' experience, family planning clinic).

*‘There are few times of shortage of some methods but we place an order for new ones and we receive them’* (Nurse, over 20 years' experience, family planning clinic).

#### Involvement of Health Care Providers in Provision of PAC

During the study, inquiries were made to ascertain whether there were adequate number of cadres involved in PAC. It was observed that physicians, nurses and midwives were all involved as a team especially in times of managing abortion complications.

*‘Both doctors and nurses are involved in PAC…. nurses are enough and normally providing counselling and contraceptive methods, except for permanent method. Yes, but we cooperate in many other things*’ (Nurse, 8 years' experience, maternity ward).

Very prominent was the lack of doctors' involvement in counselling at all the 3 facilities. Some PAC patients do not receive family planning counselling services, making PAC incomplete for such patients.

*‘Honestly, I have never seen doctors offering family planning counselling…. maybe he/she tells the woman in short like there are family planning services and if you [patient] need you can go to the clinics for more information’* (Nurse, 2 years' experience, theatre).

## DISCUSSION

The uptake of post-abortion modern family planning was low as observed in this study, 31.2%. The main factor that contributed to low uptake was inadequate counselling provided during PAC. It was observed that more than half of the participants (56%) were not counselled. The study also observed that women who received counselling had a very high uptake of family planning methods (71%).

Limited provision of family planning counselling during PAC led to missed opportunity of improving contraceptive use in this setting as well as missed opportunity to avert short inter-pregnancy intervals (< 6 months) and associated morbidities. The low uptake of post-abortion modern family planning witnessed in this study is inconsistent with other studies done in Tanzania, Kenya and in other countries in Asia and Sub Saharan Africa where 73-86% of PAC women adopted a modern family planning method.^18,20,28^ Low PAC family planning uptake has been reported in Brazil by where only 8.8% of women received family planning before hospital discharge.^17^

The reason for the low uptake of family planning in this study is evident due to the lack of proper family planning counselling provision as more than half (56%) of women did not receive family planning counselling. Other reasons for poor PAC uptake which are also mentioned in studies conducted elsewhere include lack of proper integration of PAC services with other reproductive health services. In this study, out of 69 women who were counselled and showed intention to use family planning, 11 of them could not be provided with the method of choice upon discharge due to poor coordination among HCPs. Other reasons for these inadequacies were also observed from the emerged themes in the qualitative findings obtained in all the facilities. These included; lack of skills, few providers and lack of proper coordination and organisation in PAC service delivery. In-service training of service providers on general PAC, counselling, skills in the provision of family planning methods and change of attitudes is needed in this setting. In studies where high family planning uptakes was recorded, the common denominator was comprehensive PAC and targeted family planning counselling by a dedicated team.^8,11^

Quality of family planning counselling provided during provision of PAC family planning services is also lacking. It emerged in the qualitative findings, PAC women reported that they needed more information from their HCPs during counselling. This could also explain the low uptake of modern family planning methods even among those who received counselling, when compared to findings from other studies conducted in other developed and developing countries that have linked proper family planning counselling with increased uptake of family planning up to 97% among women attending for PAC.^8,11,12^

Nearly 2/3 (63%) of those who accepted a family planning method, selected a long-term one (implant or IUCD).

Further 12% selected injectables, making 75% of them prefer a method that confers long protection with a low probability of failure. This observation is not comparable to the findings in studies in other developing countries where short-term family planning methods dominate during PAC.^17,18,28^ High uptake of long-term reversible family planning methods was observed similar to the observation made in a study conducted in the USA in 2010 by Secura and colleagues.^31^ The current government policy on postpartum and post-abortion use of contraceptives which targets building the capacity of HCPs at all levels to be able to provide long-acting and permanent methods of family planning could be a major contribution to what has been observed in this study.^6^

In contrast to observations made in a study by Prata et al. in 2011, single, divorced or widowed women were more inclined to opt for post-abortion care modern family planning methods compared to married women.^32^. This is a demonstration of decision-taking power and seeking partners support by married or cohabiting women over their reproductive health issues. On the other hand, nonuse of PAC modern family planning by women, especially those who have had previous multiple deliveries is a concern. Studies have reported that higher maternal age, shorter inter-pregnancy intervals and advanced parity are determinants of induced abortion.^33^ In countries like Tanzania where abortion is highly restricted by law, women usually resolve into unsafe abortion and are prone to lethal complications. This occurrence of non-use of family planning could be due to fear of side effects and infertility myths and misconception on modern family planning methods and poor reception by health care providers developed over time.^34,35^

Many factors were identified among health care providers that negatively affect the provision of PAC modern family planning such as inadequate skills, lack of coordination and integration of PAC with other reproductive health services. According to the HCPs, the availability of modern family planning commodities was not a barrier to the provision of PAC in the present study. These current observations further support Tanzania policy on the family planning agenda, ensuring supplies of commodities in all health facility levels to strengthen provision of family planning services.^6^

The strength of this study is in mixed-methods employed which quantify and qualify the experience of the participants. There is limited recall bias since information of interest was gathered immediately at discharge. The study enrolled both health care providers and patients, therefore experiences of both stakeholders on barriers to PAC modern family planning were equally appreciated. However, the study has to be interpreted in light of some limitations. Since this was a facility-based study and conducted among women in rural area, and in only 3 health facilities, the findings may not be generalisable to the whole population. The study looked at the uptake of family planning methods upon discharge from the facility, therefore it does not necessarily ensure the use of the method after discharge from the facility.

The facilitators to PAC uptake of modern family planning were not investigated on the qualitative arm of the study, this might add to the limitation of the study as it would have contributed to better understanding of PAC modern family planning acceptability.

## CONCLUSION

Suboptimal provision of PAC modern family planning counselling in this setting is a major bottleneck contributing to low uptake of PAC modern family planning methods. Marital status and partner's support were important predictors of PAC modern family planning uptake. However, misconception and misinformation from communication and interaction in the community are great barriers to PAC modern family planning uptake. Although the study findings revealed reported adequate supply of modern family planning commodities in all facilities, inadequate skills and lack of coordination in service delivery by the health care providers were among the challenges to the uptake of modern family planning.

There is urgent need to improve PAC family planning services through provision of regular training of health care providers on family planning counselling with comprehensive information targeting the local contextual misconception in order to attribute to reducing unsafe abortion and maternal deaths. Also, couple counselling and provision of PAC as a one-stop service should be encouraged at every facility to improve uptake and use of family planning following PAC. The Ministry of Health should emphasise the integration of reproductive health services in every health facility to avoid missed opportunities to the provision of valuable services such as family planning during PAC.
